# A Model Immunization Programme to Control Japanese Encephalitis in Viet Nam

**Published:** 2015-03

**Authors:** Nguyen Thu Yen, Wei Liu, Hoang Duc Hanh, Na Yoon Chang, Tran Nhu Duong, Robert V. Gibbons, Florian Marks, Nghiem Anh Thu, Nguyen Minh Hong, Jin Kyung Park, Pham Anh Tuan, Ananda Nisalak, John D. Clemens, Zhi-yi Xu

**Affiliations:** ^1^National Institute of Hygiene and Epidemiology (NIHE), Hanoi, Viet Nam; ^2^International Vaccine Institute, San 4-8, Bongcheon-7 dong, Kwanak-ku, Seoul 151-818, Republic of Korea; ^3^Preventive Medicine Center, HaTay Province, Viet Nam; ^4^Armed Forces Research Institute of Medical Sciences, Bangkok, Thailand; ^5^ Department of Epidemiology, Fudan University School of Public Health, Shanghai, China

**Keywords:** Demonstration project, Effectiveness study, Immunization, Japanese encephalitis, Vaccines

## Abstract

In Viet Nam, an inactivated, mouse brain-derived vaccine for Japanese encephalitis (JE) has been given exclusively to ≤5 years old children in 3 paediatric doses since 1997. However, JE incidence remained high, especially among children aged 5-9 years. We conducted a model JE immunization programme to assess the feasibility and impact of JE vaccine administered to 1-9 year(s) children in 3 standard-dose regimen: paediatric doses for children aged <3 years and adult doses for those aged ≥3 years. Of the targeted children, 96.2% were immunized with ≥2 doses of the vaccine. Compared to the national immunization programme, JE incidence rate declined sharply in districts with the model programme (11.32 to 0.87 per 100,000 in pre-versus post-vaccination period). The rate of reduction was most significant in the 5-9 years age-group. We recommend a policy change to include 5-9 years old children in the catch-up immunization campaign and administer a 4th dose to those aged 5-9 years, who had received 3 doses of the vaccine during the first 2-3 years of life.

## INTRODUCTION

Japanese encephalitis (JE), a mosquitoborne viral disease, is highly endemic in Asia (1,2). Most JE infections are asymptomatic, and only 1-3 per 1,000 infected show clinical symptoms, such as fever, headache, neck stiffness, stupor, coma, convulsion, and, occasionally paralysis (3). Children aged <15 years are at the highest risk (2,4). Approximately 10-30% of JE cases are fatal, and 30-50% of the survivors suffer from long-term neurological sequelae (5,6). JE immunization is effecacious and cost-effective and has led to rapid and drastic reduction of JE risk in JE-endemic countries (7).

In Viet Nam, JE remains a major cause of death and disability of children (8). A locally-produced, inactivated mouse brain-derived JE vaccine (locally-produced JE vaccine) has been introduced into the routine childhood immunization programme in selected high-risk districts since 1997. In the first year that JE vaccine was provided through the EPI in a district, all children aged 1-5 year(s) were targeted for immunization with the primary series. In the following and subsequent years, the children immunized in the previous year received a booster dose, and only those children aged one year received the primary dose series. All three doses were paediatric doses (0.5 mL/dose) and were given in winter campaigns. However, the international standard for the inactivated, mouse brain-derived JE vaccine is to administer paediatric doses (0.5 mL/dose) to children aged <3 years, adult doses (1.0 mL/dose) to children of ≥3 years, and a total of 4-5 doses are given as full schedule during childhood (7).

Since introduction of JE vaccine into the routine childhood immunization programme in selected high-risk districts in HaTay province in 1997-2000, JE incidence rate dropped from >20 per 100,000 in 1996 to around 5 per 100,000 in 1999, with the majority of cases still occurring among children aged <15 years. However, no further decline was noted during 1999-2004. To identify whether the continuous risk of JE after implementation of JE vaccination was associated with dose-schedule of JE vaccination and/or age group targeted for JE immunization, a model programme to immunize children at 1-9 year(s) of age with standard doses of JE vaccine was established. JE risk was compared among the 5 districts covered with the model programme and the other 9 districts covered with the national programme or no JE immunization programme.

The inactivated, mouse brain-derived vaccine produced by Green Cross, Korea (Green Cross JE Vaccine) was given in three standard doses: paediatric doses (0.5 mL/dose) for children aged <3 years and adult doses (1.0 mL/dose) for those aged 3-9 years. The first two doses were given to children at a 2-week interval; the third dose was administered a year later.

## MATERIALS AND METHODS

### Study area

HaTay, a suburb of Hanoi, was selected for the model programme. There are 14 districts in HaTay. Each is composed of 15-20 communes, with a total rural population of 2.4 million. A majority of farmers made a living from rice cultivation and pig rearing. Accessible and affordable healthcare network consisted of commune health centres, district hospital, and provincial or national hospitals. Both EPI vaccines and ‘free-market’ vaccines were delivered through and documented at commune health centres.

**Figure 1.**
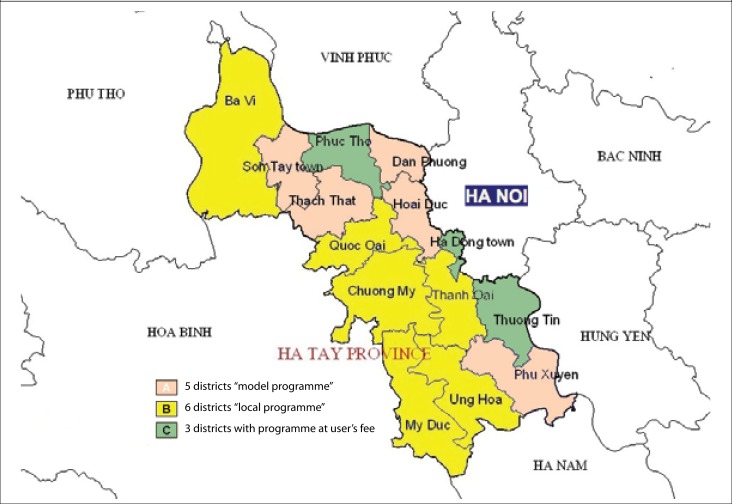
Map of HaTay province, Viet Nam

The model programme was implemented in 5 of the 14 districts for demonstration in 2004 (Group A) (Figure 1). Of the other 9 districts, 6 had introduced the locally-produced JE vaccine into the routine childhood immunization in 1997-2000 (Group B) (Figure 1); the remaining 3 districts had no JE vaccination programme (Group C). ‘Free-market’ JE vaccine had been available in all 14 districts at US$ 1-2 per dose since 1995.

### Household survey of EPI logbooks in the 5 demonstration districts

A ‘door-to-door’ household survey was conducted in the Group A districts to record all children born after November 1994 as well as to ascertain their JE vaccination history by checking the pre-existing immunization log books at the community health centres and/or immunization card at homes. Those who had no documented previous JE vaccination were identified as eligible for JE immunization. Children who had an incomplete series were invited to receive their remaining doses but this group was not included in the final analysis of data. Age and names of children, immunized and not immunized, their parents’ names and addresses were logged on special forms and entered onto a computer database. Numbers of children by age from other 9 districts were obtained from EPI department of the Center for Disease Control and Prevention in HaTay.

### Training and campaign publicity

Training courses on household survey and vaccination campaign were organized for staff members of commune health centres. Meetings, radio broadcasting, and posters for campaign publicity were initiated 1-2 week(s) prior to each round of JE immunization. Written informed consent was obtained from parents before the first round of the vaccination campaign.

### Mass JE immunization campaign

Two hundred thirty-seven centres were prepared for the vaccination campaigns in November 2004. The third round of the campaign for a booster dose was launched in November 2005. Each round of campaign lasted for 2-3 days. Children who missed one of the 2 doses in 2004 received 2 doses in 2005.

### Hospital-based surveillance for JE

Two Provincial Hospitals in HaTay and the National Pediatric Hospital in Hanoi, which were most likely to encounter acute encephalitis patients from HaTay, were used as referral hospitals for JE surveillance from January 2004. Paediatricians from all 14 district hospitals were trained to recognize and to refer encephalitis patients to one of the 3 surveillance hospitals. Residents of HaTay, aged ≤15 years, with a clinical diagnosis of acute encephalitis syndrome (AES), were enrolled as suspected JE cases. Cerebrospinal fluid (CSF) was collected at admission in hospital. Presence of IgM antibody to JEV (anti-JEV) in cerebrospinal fluid (CSF) was defined as JE (9,10). Initial testing of specimens was performed at the National Pediatric Hospital Laboratory and Laboratory of HaTay Center for Preventive Medicine; confirmatory testing of all specimens was done at the Virology Laboratory of Armed Forces Research Institute of Medical Sciences (AFRIMS), Bangkok, Thailand. A monthly contact with paediatric infectious diseases departments and emergency departments at the 3 surveillance hospitals was made to check the number of patients with acute encephalitis and those diagnosed to have JE.

### Vaccination coverage

Children who received JE vaccine through the model immunization programme in the Group A districts were recorded and entered into a database. Data on the coverage in Group B districts, where JE vaccine was delivered through routine EPI or in Group C districts with ‘free-market’ JE vaccine, were obtained from immunization logbooks that were maintained at the commune health centres.

### Impact of the model programme on risk of JE

To assess the impact of the model immunization programme, annualized age-specific JE incidence rates were compared post-versus pre-vaccination campaign. The Wilcoxon Rank-Sum test was used for the analysis of rate differences of post-versus pre-campaign between the demonstration and non-demonstration districts. Computerized database of vaccination records was used for validation of vaccination history of each confirmed JE case in the demonstration districts. In non-demonstration districts, JE vaccination history among confirmed JE patients were assessed by checking routine immunization logbooks at commune health centres and/or individual immunization card at home.

### Data management and data analysis

A structural data management system using Microsoft FoxPro^®^ 2000 was designed for the study. All data were double-entered. Dual checks and error checks were performed on all batches of data to assure validity, integrity, and confidentiality of data. Data were analyzed with SAS statistical software 8.1 (SAS Institute Inc., Cary, NC, USA).

### Ethical clearance

The study was approved by the Institutional Review Boards (IRBs) at the National Institute of Health and Epidemiology (NIHE), Viet Nam, and the International Vaccine Institute (IVI), Seoul. Confidentiality of each participant was ensured, and any possible concern was discussed prior to the start of the project.

## RESULTS

### Vaccination coverage

Of 114,858 children aged 1-9 year(s) in the Group A districts, 109,902 (95.7%) were visited during the door-to-door household surveys; the remaining 4,956 (4.3%) were absent and could not be included in the immunization programme. Of the 109,902 children, 47,850 (43.5%) had received ≥2 doses of ‘free-market’ JE vaccine; thus, they were not eligible for participating in the model immunization programme. The remaining 62,052 (56.5%) children without history of JE immunization were enrolled in the model immunization programme. Assuming that, among the 4,956 children who were missed in the course of the household survey, a similar proportion has been vaccinated previously through the ‘free market’ as in the surveyed group, 2,800 (4,956×56.5%) children were estimated as unvaccinated.

Of the 62,052 children, 60,893 (98.1%) received the first dose of Green Cross JE Vaccine; 60,531 (97.5%) returned for the second dose, and 60,165 (96.9%) were immunized with full three doses of the Green Cross JE Vaccine; 1,159 children declined the JE vaccine. Hence, of the 114,858 children, 4,321 were estimated as unvaccinated or received a single dose. The coverage with at least two doses of the Green Cross JE Vaccine through the model programme was, thus, estimated at 96.2% for the Group A districts.

In the Group B districts, children aged 1-5 year(s) should have been immunized with locally-produced JE vaccine from the EPI during 1997-2000 programme. Subsequent birth cohorts were immunized at 12-23 months of age during the annual JE vaccination campaign. By December 2004, children aged 1-9 year(s) in the Group B districts would have been vaccinated with the locally-produced JE vaccine. Based on the previous EPI documents, JE vaccine coverage in this age-group was 95.8% on average.

### Severe adverse events (SAE) following vaccination

One case with fever (39 °C) and rash was reported during the mass vaccination campaigns. The disease was self-limiting. The sick child was hospitalized and discharged one day later.

### JE incidence

From January 2004 through December 2006, 110 acute encephalitis cases were reported; 75 (68.2%) were confirmed as JE via positive JEV IgM ELISA test in their CSFs, and 50 JE cases belonged to the 2004 cohorts aged 1-9 year(s) whose incidence rates were 11.32, 2.15, and 2.79 per 100,000 in Group A, B and C districts respectively in 2004 (Table 1) and were 5.22, 7.51, and 9.75 per 100,000 respectively in 2005; the rates declined significantly in all 3 groups in 2006. Thus, the annual incidence rate sharply declined for Group A and significantly increased for Group B and C in 2005 (Table 1). The impact of the 2004 model immunization programme on JE risk in Group A districts was, thus, well-demonstrated.

**Figure 2.**
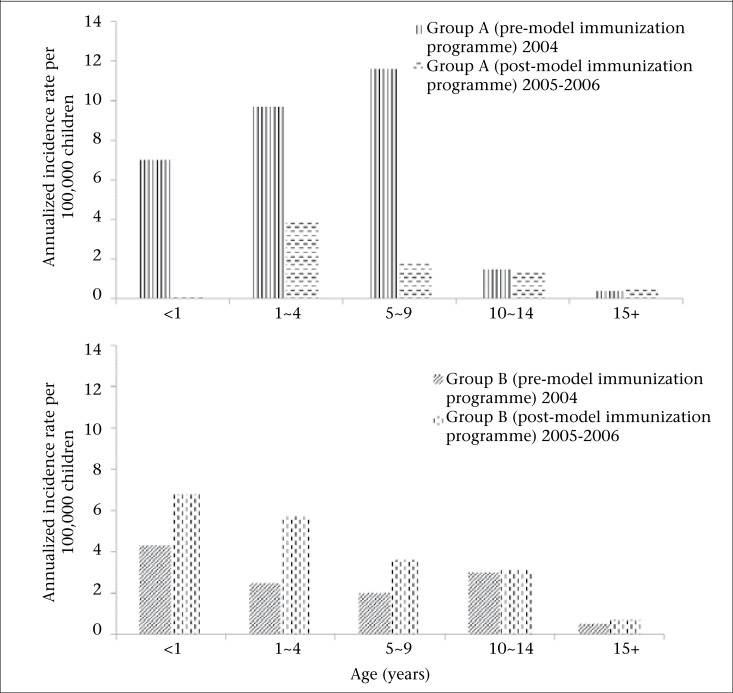
Annualized JE incidence rates by age and by programme, HaTay, Viet Nam, 2004-2006

In the Group A districts, JE rate peaked in 5-9 years age-group before implementation of the model programme in 2004; it drastically dropped for this age-group in 2005-2006 (Figure 2). In Group B districts, JE incidence rate peaked in children younger than 4 years of age because children younger than 23 months of age might not have received JE vaccine during annual winter campaigns.

### Impact of the model programme on risk of JE

The Wilcoxon Rank-Sum test (11) was used for testing the rate difference in the post-(2005-2006) versus pre-(2004) campaign between the demonstration and non-demonstration districts. For each district, a rate difference was defined as post-campaign rate minus pre-campaign rate, and a rank of the rate difference was given to all 14 districts. Of the 9 non-demonstration districts, six reported a rate increase while the five demonstration districts all showed a rate decrease (Table 2). The Wilcoxon test showed statistically significant results in comparing the rate differences of the five demonstration districts versus the nine non-demonstration districts (p<0.05) as well as the six Group B districts (p<0.05) (Table 2). The impact of the model programme on JE risk was, therefore, higher than of the national programme.

**Table 1.** JE incidence rate (IR) per 100,000 in 2004 cohort of 1-9 year(s) covered with different JE immunization programme, 2004-2006*

Group	District	No. of children [1-9 year(s)]	JE cases 2004 (IR)	JE cases 2005 (IR)	JE cases 2006 (IR)
A	5 districts with model programme	114,858	13 (11.32)	6 (5.22)	1 (0.87)
B	6 districts with local EPI programme	186,417	4 (2.15)	14 (7.51)	1 (0.54)
C	3 districts with programme at user-fee (free-market vaccine)	71,793	2 (2.79)	7 (9.75)	2 (2.78)

*In 2004, JE vaccine covered 1-9 year(s) old children only in the 6 districts of Group B; it was introduced into Group A during December 2004−January 2005

IR=Incidence rate (per 100,000)

Of the seven JE cases, aged 1-9 year(s), from the 5 demonstration districts (Group A districts) in 2005-2006 (Table 2), five (5/4,321) had belonged to un-immunized group, one had received 2 doses from the model programme in 2004 (1/60,531), a reduction of 98.5% in the vaccine group (p<0.001). Another case had received full 3 doses of JE vaccine from the ‘free market’ (1/47,850) in 2002/2003. No single JE case was detected among the 60,165 children who had received full three doses of the vaccine from the model immunization programme. Six JE cases aged 6-12 months (who had not been targeted for JE vaccination) were found in the Group B districts. Also, four JE cases were found in children between 13 and 23 months of age; these four children had not been immunized as they were born 1-11month(s) before the winter campaign and, thus, were not included in that respective campaign.

## DISCUSSION

The model programme of JE immunization was well-received by parents, communities, public health professionals, and the Government; high coverage with full three doses of the vaccine was achieved through the current healthcare infrastructure in rural Viet Nam. The direct cost of training, household surveys, staff salary, and other renewable materials for vaccination campaign was only US$ 0.22 per dose. The time required for each round of campaign was only 2-3 days. Therefore, immunization of 1-9 year(s) old children, with three standard doses, was feasible and acceptable.

**Table 2.** JE incidence rates among children aged 1-9 year(s) in post-(2005-2006) versus pre-(2004) vaccination period in 14 districts of HaTay province, Viet Nam

District	Group	JE cases in pre-vaccination period (incidence rate, per 100,000)	JE cases in post-vaccination period (incidence rate, per 100,000)	Risk difference	Rank*
Son Tay	A	3 (19.55)	0 (0)	-19.55	2
Hoai Duc	A	2 (6.68)	2 (3.34)	-3.34	6
Dan Phuong	A	5 (25.30)	2 (5.06)	-20.24	1
Thach That	A	2 (8.10)	2 (4.05)	-4.05	5
Phu Xuyen	A	1 (3.98)	1 (1.99)	-1.99	7
Ba Vi	B	0 (0)	3 (4.30)	4.30	12
Quoc Oai	B	0 (0)	0 (0)	0.00	8
Chuong My	B	1 (2.35)	4 (4.71)	2.35	10
Thanh Oai	B	1 (3.19)	4 (6.37)	3.19	11
Ung Hoa	B	2 (6.36)	1 (1.59)	-4.77	4
My Duc	B	0 (0)	3 (5.99)	5.99	13
Phuc Tho	C	0 (0)	1 (2.03)	2.03	9
Thung Tin	C	1 (3.06)	8 (12.22)	9.17	14
Ha Dong	C	1 (6.94)	0 (0)	-6.94	3

*Wilcoxon Rank Sum Test: p=0.0329 Group A vs Group (B+C)

p=0.0358 Group A vs Group B

The difference in the trend of JE risk between the 5 demonstration and the 9 non-demonstration districts, especially between Group A and B districts, was statistically significant. The higher impact of the model programme compared to the national programme on the JE risk reduction cannot be explained by different vaccine coverage, which was 95.8% for the Group B districts and 96.2% for the Group A districts. The drastic decline of JE incidence rate in Group A districts was due to effective immunization of most susceptible children immediately before the epidemic year 2005; the rate increase in the Group B districts in 2005 was possibly associated with the waning immunity or inadequate protection of the three-paediatric dose regimen for older children. The use of different JE vaccines in the various districts was a limitation of the study. However, these vaccines are produced by different manufactures using the same technology. A recent study found that the effectiveness of the Vietnamese JE vaccine was similar to that reported in Thailand and Taiwan where the same vaccine was delivered but a total of 4 or 5 doses were administered to children through routine immunization programme (12). To avoid the waning immunity or the inadequate protection conferred by the three-paediatric dose regimen, we recommend a fourth dose at 5-9 years of age as was done routinely in Japan, Korea, and Taiwan, where the same JE vaccine was used, and their JE immunization programmes were more successful in the control of JE (13–15). It is important to note that inclusion of 5-9 years old children in the vaccination campaign has increased the impact of the model programme.

In the Group B districts, JE incidence rate in the 10-14 years age-group was similar to or higher than the rate in 5-9 years age-group (Figure 2). Had the local JE immunization programme been targeted also to those children who were 5-9 years of age during 1997-2000 as we did in the current model programme, the rate in the 10-14 years age-group in 2004-2006 would have been reduced considerably. Therefore, we recommend the inclusion of 5-9 years age-group in the catch-up JE immunization campaign. Analysis of the situation and recommendations for modification of the national JE immunization programme were discussed in another article.

In this study, we had no randomly-allocated control group for comparison of JE risk. In spite of the fact that all 14 districts in HaTay were close neighbours in the same suburb of Hanoi, the decreased JE rate post-versus pre-campaign in the Group A districts and the increased rate in the Group B districts could be partially due to variation of JE risk by year and place. In addition, there are no sufficient data to assess the effectiveness of the three paediatric doses because only 15% of the immunized children in the Group B districts received JE vaccine at 3-5 years of age. Longer follow-up and further study are necessary to look into these issues. Finally, unlike in Group A districts where we estimated the vaccine coverage rate by using our field survey data, in Group B and C districts, we directly used the government data as a basis for vaccine coverage estimate, which may introduce some measurement bias.

The seroconversion and geometric mean titre (GMT) of the neutralizing antibody to the locally-produced JE vaccine was almost the same as, if not better than, the inactivated mouse brain-derived JE vaccine produced by the Biken Institute, Japan. So far, no conclusion can be made that the effectiveness of the Green Cross JE Vaccine was different from the local JE vaccine which costs only US$ 0.15 per paediatric dose, and the vaccine production has been increasing steadily. If the policy can be changed to include 5-9 years old children in the catch-up campaign and administer the 4th dose to those of 5-9 years old children who had received three paediatric doses through the national JE immunization programme, the goal of JE control in Viet Nam could be a reality in the near future.

## ACKNOWLEDGEMENTS

Financial support for this project was provided by the Korean International Cooperation Agency (KOICA) by contract no. 2003-001. The authors thank colleagues from HaTay Provincial Hospital, and Hanoi National Pediatric Hospital for their participation and contribution in the investigation. We thank doctors working at the commune health centres in HaTay for their help with the mass immunization campaign.

**Conflict of interest:** Authors declare no competing interests.
